# Numbers of mutations to different types of colorectal cancer

**DOI:** 10.1186/1471-2407-5-126

**Published:** 2005-10-03

**Authors:** Peter Calabrese, Jukka-Pekka Mecklin, Heikki J Järvinen, Lauri A Aaltonen, Simon Tavaré, Darryl Shibata

**Affiliations:** 1 Program in Molecular and Computational Biology, Department of Biological Sciences, University of Southern California, Los Angeles, CA 90089, USA; 2 Second Department of Surgery, Helsinki University Central Hospital, FIN-00029, Helsinki, Finland; 3 Jyvaskyla Central Hospital, FIN-40620, Jyvaskyla, Finland; 4 Department of Medical Genetics, Haartman Institute, FIN-00014, University of Helsinki, Finland; 5 Department of Biological Sciences, University of Southern California, Los Angeles, CA 90089, USA, and Department of Oncology, University of Cambridge, Cambridge, UK; 6 Department of Pathology, University of Southern California School of Medicine, Los Angeles, CA 90033, USA

## Abstract

**Background:**

The numbers of oncogenic mutations required for transformation are uncertain but may be inferred from how cancer frequencies increase with aging. Cancers requiring more mutations will tend to appear later in life. This type of approach may be confounded by biologic heterogeneity because different cancer subtypes may require different numbers of mutations. For example, a sporadic cancer should require at least one more somatic mutation relative to its hereditary counterpart.

**Methods:**

To better estimate numbers of mutations before transformation, 1,022 colorectal cancers were classified with respect to microsatellite instability (MSI) and germline DNA mismatch repair mutations characteristic of hereditary nonpolyposis colorectal cancer (HNPCC). MSI- cancers were also classified with respect to clinical stage. Ages at cancer and a Bayesian algorithm were used to estimate the numbers of oncogenic mutations required for transformation for each cancer subtype.

**Results:**

Ages at MSI+ cancers were consistent with five or six oncogenic mutations for hereditary (HNPCC) cancers, and seven or eight mutations for its sporadic counterpart. Ages at cancer were consistent with seven mutations for sporadic MSI- cancers, and were similar (six to eight mutations) regardless of clinical cancer stage.

**Conclusion:**

Different biologic subtypes of colorectal cancer appear to require different numbers of oncogenic mutations before transformation. Sporadic MSI+ cancers may require more than a single additional somatic alteration compared to hereditary MSI+ cancers because the epigenetic inactivation of MLH1 commonly observed in sporadic MSI+ cancers may be a multistep process. Interestingly, estimated numbers of MSI- cancer mutations were similar (six to eight mutations) regardless of clinical cancer stage, suggesting a propensity to spread or metastasize does not require additional mutations after transformation. Estimates of oncogenic mutation numbers may help explain some of the biology underlying different cancer subtypes.

## Background

Cancer is thought to arise through a multistep process involving sequential cycles of mutation and selection [1]. The identities and numbers of mutations required for transformation are uncertain, but perhaps six general cellular functions are typically altered [2]. Numbers of oncogenic mutations may also be inferred from the age-related increases in frequencies observed with many cancer types. For example, logarithms of cancer frequencies versus age typically yield straight lines, with slopes proportional to numbers of cancer mutations [3].

Colorectal cancer epidemiology is consistent with approximately five to seven oncogenic mutations before transformation [3-6]. The variability in estimated numbers of mutations may reflect a number of differences. For example, estimates vary between populations, with five to six mutations in England and six to seven mutations in Finland [4]. Recent advances in cancer genetics also reveal biologic colorectal cancer heterogeneity. Approximately 5% of all colorectal cancers have strong familial predispositions and arise in individuals with germline mutations in critical susceptibility loci [7]. Such hereditary cancers (familial adenomatous polyposis (FAP) and hereditary nonpolyposis colorectal cancer (HNPCC)) typically present at younger ages and should require fewer somatic mutations than their sporadic counterparts because one mutation is inherited.

Genetic instability also divides colorectal cancers into two groups [8]. Approximately 10 to 15% of sporadic cancers exhibit microsatellite instability (MSI) secondary to somatic loss of DNA mismatch repair (MMR). Most other cancers exhibit chromosomal instability (CIN) characterized by aneuploidy and loss of heterozygosity (LOH) [7,8]. CIN and MSI+ colorectal cancers have different characteristics with respect to mutated loci, tumor location, morphology, and clinical outcomes [7,8].

Numbers of oncogenic mutations may differ between cancer subtypes. Therefore, colorectal cancers arising in a population-based setting were molecularly classified as either sporadic or hereditary, and MSI+ or MSI-. Cancers were also classified with respect to clinical stage because additional mutations may be required for invasion or metastasis. Ages at cancer for each subgroup were used to infer numbers of mutations required for each type of colorectal cancer.

## Methods

### Specimens

MSI status was determined for 1,022 colorectal cancers sampled from nine large regional hospitals in southeastern Finland as part of a study to characterize genetic alterations in a well-defined population [9]. The cancers represent approximately 60% of all colorectal cancers removed from this population in 1994 to 1998 [9]. Germline mutations in MLH1 or MSH2 were detected by allelic specific PCR assays (for the two common Finnish MLH1 germline mutations) or by direct genomic sequencing of coding exons [9]. The data can be downloaded from the following website: . Approval for this research was obtained from the appropriate ethics committees, which are in compliance with the Helsinki Declaration.

A second data set (SEER 11 Regs Public-Use, Nov 2001 Sub (1992–1999)) was obtained from the Surveillance, Epidemiology, and End Results (SEER) Program, a population-based registry in the United States of America that records all cancers regardless of clinical treatment [10]. A total of 108,275 records were analyzed for ages at cancer selected by site (colon and rectum), race (white), histology (adenocarcinoma, ICD-0-2 codes 8000–8500), and stage (localized, regional, or distant). These cancers were not characterized with respect to HNPCC or MSI.

### Quantitative analysis

Numbers of oncogenic alterations (genetic mutations or epigenetic alterations) required for transformation were estimated from ages at cancer using a Bayesian approach as previously described [11]. This method requires the use of a life table from census data: for the Finnish data set we used a Finnish life table from the World Health Organization website , for the SEER dataset we used a United States life table as described previously [11]. The model assumes the first visible clonal expansion occurs at the time of transformation and ignores the interval after transformation. The analysis ignores temporal trends, which may influence our mutation estimates.

For the SEER dataset, we also fit our model for cancer progression [11] with the inferential method described in reference 12. This method does not require a life table, but unlike our method it does require information on all the cancer cases for the population at risk. Therefore this method is appropriate for analysing the SEER dataset but not the Finnish dataset. Our method [11] is appropriate for analysing both datasets. For the SEER dataset, the two methods inferred the same number of mutations required for cancer.

## Results

The presence or absence of MSI was determined for 1,022 colorectal cancers obtained from nine large regional hospitals in southeastern Finland [9]. There were 895 (87.6%) MSI- cancers and 127 (12.4%) MSI+ cancers. The MSI+ cancers were further classified as sporadic (N = 98 or 9.6% of all cancers) or HNPCC (N = 29 or 2.9% of all cancers) based on germline MLH1 or MSH2 mutations (Table 1).

**Figure 1:**
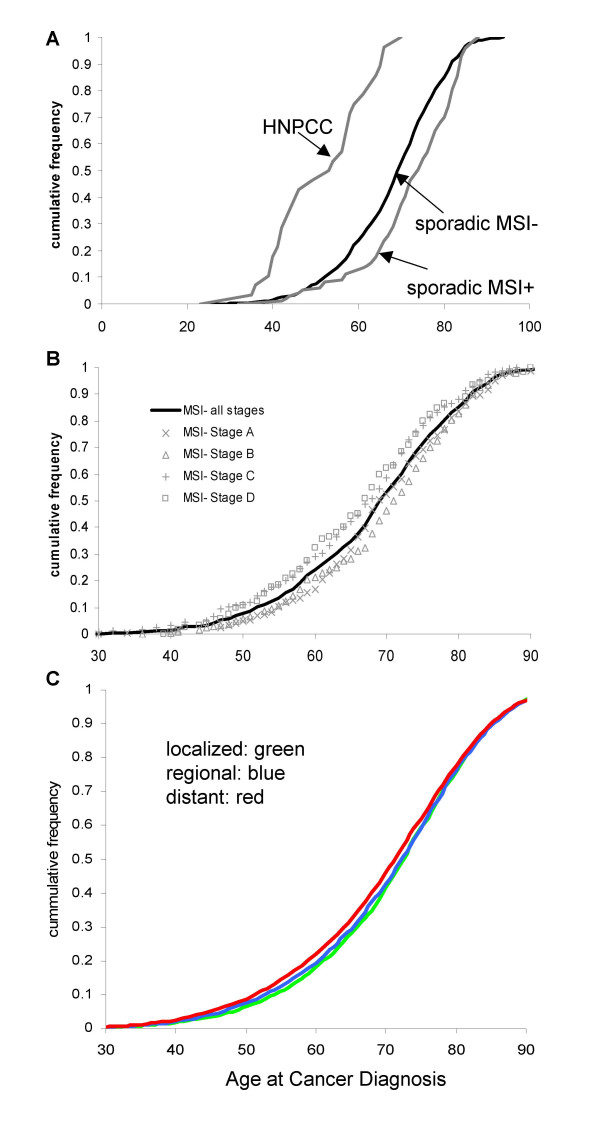
**Cumulative colorectal cancer frequencies and patient ages at diagnosis**. A) Finnish MSI-, and hereditary (HNPCC) and sporadic MSI+ colorectal cancers. B) Finnish MSI- cancers with respect to Duke's stage. C) SEER data with respect to clinical stage.

Ages at cancer can be used to estimate likely numbers of oncogenic mutations required before transformation [3-6,11]. Average ages for sporadic MSI+, MSI-, and HNPCC cancers were respectively 71.5, 67.5, and 50.3 years (Figure 1A). For HNPCC cancers, estimated numbers of oncogenic mutations were between four and seven (95% credibility interval), with the most likely value of five mutations (Table 1). For MSI+ sporadic cancers, estimated numbers of mutations were between six and nine (95% credibility interval) with more likely values of seven or eight mutations. The most likely number of mutations was seven for sporadic MSI- cancers.

Duke's stage and age at clinical presentation (Figure 1B) were documented for 884 of the 895 MSI- sporadic cancers (Table 1). Average ages were 68.6 years for stage A, 69.0 years for stage B, 65.2 years for stage C, and 65.4 years for stage D. The most likely numbers of oncogenic mutations were seven for stage A cancers, eight for stage B cancers, and six for stage C or D cancers (Table 1).

Mutation number estimates with respect to clinical stage may be biased with the Finnish data because it includes only specimens with tissue available for molecular analysis. Advanced cancers may not be removed. Therefore, a similar analysis was performed on a population-based cancer registry [10] from the United States of America (SEER 11 Regs Public-Use, Nov 2001 Sub (1992–1999)), which records ages and stages at diagnosis regardless of treatment (Table 2). The average age at diagnosis was 70.5 years, consistent with an estimate of six mutations to colorectal cancer for the 108,275 white males and females with stage data. Like the Finnish cancers, ages were similar for SEER patients of different clinical stages, with an estimate of six mutations for cancers with localized, regional or distant clinical stages (Table 2 and Figure 1C).

## Discussion

The exact identities and numbers of mutations required for transformation are uncertain. With simple multistage models [3-6,11], all cancers of a given type require the same number of oncogenic mutations, but stochastic differences in the times to accumulate these mutations allow individual cancers to appear at different ages. Precisely when and how quickly mutations accumulate are unknown, but a basic premise is that cancer types requiring more mutations will tend to appear later in life. Therefore, numbers of mutations may be estimated from cancer epidemiology. Colorectal cancer frequencies increase with age, and the pattern of this increase is consistent with approximately five to seven oncogenic mutations [3-6].

In this study numbers of mutations were estimated for well-defined subgroups of colorectal cancers because biological heterogeneity may confound this type of quantitative analysis. Such estimates should be considered rough guides rather than absolute values because our model does not account for all factors. Cancers were classified as MSI+ or MSI-, and MSI+ cancers were further sub-classified as either hereditary (HNPCC) or sporadic. As expected because one MMR mutation is inherited, estimated numbers of critical mutations were less for MSI+ HNPCC cancers compared to sporadic MSI+ cancers. However, sporadic MSI+ cancers required more than one additional somatic mutation compared to HNPCC cancers. Of interest, a difference of more than a single mutation has also been inferred between sporadic and FAP cancers, with estimates of three to four mutations for FAP cancers versus six for sporadic cancers [6,13], although another analysis was consistent with a difference of only a single mutation [14]. Therefore, germline mutations (APC and MMR loci) in both common colorectal familial cancer syndromes (FAP and HNPCC) appear to advance progression by more than a single mutation relative to their sporadic counterparts.

An epigenetic mechanism may help explain why sporadic MSI+ cancers require more than one additional somatic alteration relative to HNPCC cancers. Inactivation of the normal MMR allele occurs through mutation (usually LOH [15]) in HNPCC whereas MMR loss in sporadic MSI+ cancers is associated with MLH1 promoter methylation [16,17]. CpG islands may be "protected" from methylation because most are unmethylated at birth and usually remain unmethylated throughout life [18]. Epigenetic MLH1 inactivation may require at least two cis acting somatic alterations---loss of a mechanism that normally prevents methylation, followed by the accumulation of methylation at sufficient numbers of CpG sites to silence expression.

In agreement with prior studies, there were seven mutations estimated for sporadic MSI- Finnish cancers [4], and seven or eight mutations for MSI+ cancers. A requirement for more alterations before tranformation for sporadic MSI+ compared to sporadic MSI- cancers may help explain why sporadic MSI+ cancers are a minority of all colorectal cancers and occur in slightly older patients [19,20]. Although numbers of oncogenic mutations before transformation are similar between sporadic MSI+ and MSI- cancers, their identities likely differ [7,8].

**Figure 2:**
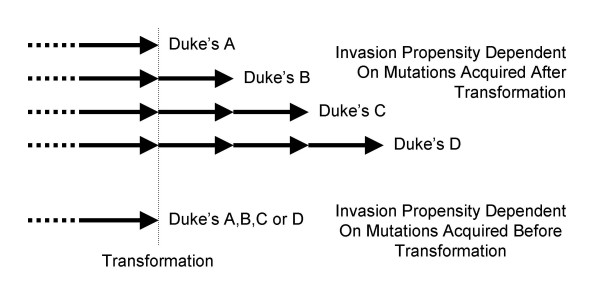
**Mutation timing and numbers with respect to invasiveness**. If invasion or metastasis depends on mutations acquired after transformation, clinically higher stage cancers would be expected to require more time and mutations. However, if an invasive phenotype depends on mutations acquired before transformation, cancers of different clinical stages could require similar numbers of oncogenic mutations and times for progression.

Colorectal cancers also differ by their extent of spread. Progression to metastasis may involve a long sequence of potentially rate limiting steps [21]. If invasion or metastasis depends on mutations that arise after transformation, advanced cancers should require more oncogenic mutations and more time for progression (Figure 2). However, ages at diagnosis and estimated mutation numbers did not markedly differ between cancers of different clinical stages.

Equivalent numbers of mutations regardless of clinical stage are consistent with recent speculation that an invasive potential is acquired early in progression [22], albeit only rare cells actually form visible metastases. Primary breast cancer expression patterns correlate with clinical outcomes or metastases [22-25], suggesting that a propensity to spread is already present at the time of transformation. Alternatively, all cancers may have the same abilities to invade and metastasize, with clinical stage dependent on random events that occur rapidly after transformation. A short interval between transformation and detection may help limit spread because clinical surveillance tends to detect localized colorectal cancers [26-28].

**Figure 3:**
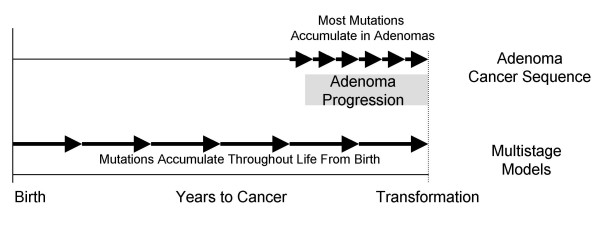
**Multistage versus tumor progression models**. In multistage models, early mutations fail to confer visible changes in phenotype because they accumulate throughout life from birth. Tumors appear only after a cell has accumulated a critical number or combination of oncogenic mutations. In contrast, the adenoma-cancer sequence suggests most oncogenic mutations confer stepwise incremental changes in phenotype and accumulate much later in life in visible tumors.

Multistage models are mechanistically different from tumor progression models and more consistent with a hypothesis that mutations acquired early during progression help determine extent of invasion (Figure 3). Mutations sequentially accumulate before transformation in both models, but the adenoma-cancer sequence suggests most cancer mutations start to accumulate after the age of 50 years in adenomas [7]. Such tumor progression imposes purpose to early mutations because each additional mutation confers incremental changes to a non-invasive adenoma phenotype. Therefore, tumor progression models would likely differ between MSI+ and MSI- cancers because their biology and types of mutations are quite different [7,8].

In contrast, mutations accumulate throughout life in multistage models. Genetically engineered mice and familial cancer syndromes reveal that many oncogenic mutations are also compatible with normal phenotypes [11], allowing for the possibility that many "cancer" mutations may first accumulate in normal-appearing colon very early in life. Such pretumor progression [11] more readily allows for an invasive or metastatic cancer phenotype at transformation because genetic progression is uncoupled from tumor progression (Figure 2). Rather than incremental stepwise changes in phenotype after each new mutation, a tumor phenotype may only emerge after several initially occult mutations accumulate in a single normal appearing cell. In this way our multistage model can apply to both MSI+ and MSI- cancers despite their marked differences in types of mutations because early critical mutations (whatever they are) do not visibly change phenotype but instead accumulate in normal appearing colon. Early or advanced sporadic MSI- colorectal cancers appeared to require similar numbers of mutations, consistent with the phenotype at cancer diagnosis contingent on mutations acquired much earlier in life and present at the time of transformation. However, ascertainment bias may also be responsible for the similar frequency-age distributions of colorectal cancers of different clinical stages.

Progression to cancer has been modeled by a number of investigators with different approaches and assumptions [3-6,11-14,29,30]. In our previously reported approach there is no growth until after the last required mutation has been acquired [11]. In this paper we apply this model to cancer subtypes instead of considering colorectal cancers as a single uniform disease. Modeling is potentially more informative and specific when applied to distinct cancer subtypes because their progression pathways can differ. The ability to apply a simple multistage model to different colorectal cancer subtypes that have marked differences in final types of mutations and clinical outcomes suggests its basic underlying premise (most critical alterations first accumulate in normal colon) may be correct.

## Conclusion

The biology of cancer must underlie the epidemiology of cancer. Here we illustrate that multistage models provide conceptually plausible solutions even when colorectal cancers are divided into biologically relevant and quite different subtypes. Ages at cancer are consistent with five or six somatic oncogenic mutations for hereditary (HNPCC) MSI+ cancers and seven or eight mutations for its sporadic counterpart. The apparent requirement for more than one additional somatic mutation in sporadic MSI+ cancers may reflect that MMR inactivation is commonly epigenetic, which may involve multiple steps. Ages at MSI- cancers were consistent with six or seven oncogenic mutations, with similar estimates for all clinical stages, suggesting that mutations acquired very early in life dictate the cancer phenotype at the time of transformation. Better integration of cancer epidemiology with its biology remains a further challenge.

## Competing interests

The author(s) declare they have no competing interests.

## Authors' contributions

Drs. Calabrese and Tavaré developed and applied the Bayesian algorithm, and edited the manuscript. Drs. Aaltonen, Mecklin, and Järvinen provided the clinical and molecular information for the Finnish cohort. Dr. Shibata helped analyze the data and wrote the manuscript.

## Pre-publication history

The pre-publication history for this paper can be accessed here:



## Figures and Tables

**Table 1:** Finnish Colorectal Cancers Sample

**Cancer Type**	**Average Age (years)**	**Number (%)**	Most Likely Numbers of Mutations*					
			
			**4**	**5**	**6**	**7**	**8**	**9**
HNPCC	50.3	29 (2.9)	0.08	0.43	0.39	0.09		
Sporadic MSI+	71.5	98 (12.4)		0.03	0.2	0.34	0.33	0.1
Sporadic MSI-	67.5	895 (87.6)			0.01	0.99		
								
Sporadic MSI-								
Stage A	68.6	187 (21.2)			0.09	0.75	0.16	
Stage B	69.0	330 (37.3)				0.44	0.56	
Stage C	65.2	246 (27.8)			0.8	0.2		
Stage D	65.4	121 (13.7)		0.06	0.66	0.28		

* Probabilities of the number of mutations required for cancer. Most likely values are underlined.

**Table 2:** SEER Colorectal Cancers

**Cancer Type**	**Average Age (years)**	**Number (%)**	Number of Mutations*
All Cancers	70.5	108,275	6
Localized	70.9	44,773 (38.7)	6
Regional	70.5	42,380 (36.6)	6
Distant	69.5	21,122 (18.3)	6

* Most likely values
